# Prevalence and clinical implications of valvular calcification on coronary computed tomography angiography

**DOI:** 10.1093/ehjci/jeaa263

**Published:** 2020-12-11

**Authors:** Michelle C Williams, Daniele Massera, Alastair J Moss, Rong Bing, Anda Bularga, Philip D Adamson, Amanda Hunter, Shirjel Alam, Anoop S V Shah, Tania Pawade, Giles Roditi, Edwin J R van Beek, Edward D Nicol, David E Newby, Marc R Dweck

**Affiliations:** 1 University of Edinburgh/British Heart Foundation Centre for Cardiovascular Science, Chancellor’s Building, 49 Little France Crescent, Edinburgh EH164SB, UK; 2 Edinburgh Imaging Facility QMRI, University of Edinburgh, Edinburgh EH164TJ, UK; 3 Montefiore Medical Center and Albert Einstein College of Medicine, Bronx, NY 10461, USA; 4 Christchurch Heart Institute, University of Otago, Christchurch 8140, New Zealand; 5 Glasgow Clinical Research Imaging Facility, Queen Elizabeth University Hospital, Glasgow G514LB, UK

**Keywords:** aortic valve, mitral valve, computed tomography, computed tomography coronary angiography

## Abstract

**Aims:**

Valvular heart disease can be identified by calcification on coronary computed tomography angiography (CCTA) and has been associated with adverse clinical outcomes. We assessed aortic and mitral valve calcification in patients presenting with stable chest pain and their association with cardiovascular risk factors, coronary artery disease, and cardiovascular outcomes.

**Methods and results:**

In 1769 patients (58 ± 9 years, 56% male) undergoing CCTA for stable chest pain, aortic and mitral valve calcification were quantified using Agatston score. Aortic valve calcification was present in 241 (14%) and mitral calcification in 64 (4%). Independent predictors of aortic valve calcification were age, male sex, hypertension, diabetes mellitus, and cerebrovascular disease, whereas the only predictor of mitral valve calcification was age. Patients with aortic and mitral valve calcification had higher coronary artery calcium scores and more obstructive coronary artery disease. The composite endpoint of cardiovascular mortality, non-fatal myocardial infarction, or non-fatal stroke was higher in those with aortic [hazard ratio (HR) 2.87; 95% confidence interval (CI) 1.60–5.17; *P* < 0.001] or mitral (HR 3.50; 95% CI 1.47–8.07; *P* = 0.004) valve calcification, but this was not independent of coronary artery calcification or obstructive coronary artery disease.

**Conclusion:**

Aortic and mitral valve calcification occurs in one in six patients with stable chest pain undergoing CCTA and is associated with concomitant coronary atherosclerosis. Whilst valvular calcification is associated with a higher risk of cardiovascular events, this was not independent of the burden of coronary artery disease.

## Introduction

Valvular heart disease is an important cause of morbidity and mortality worldwide and shares similar risk factors with coronary artery disease.^1^ Incidental calcification of the aortic or mitral valve on computed tomography (CT) may identify patients with previously undiagnosed valvular heart disease. Coronary CT angiography (CCTA) is now a widely used non-invasive imaging modality, frequently the first-line investigation for patients with suspected coronary artery disease.^2^^,^^3^ Therefore, the identification of incidental valvular calcification on CCTA represents a potentially important opportunity for screening and patient risk stratification.^4–7^

Aortic valve calcification is an established marker of aortic stenosis, with the aortic valve calcium score on CT associated with hemodynamic severity of aortic stenosis on echocardiography.^8–10^ Robust sex-specific aortic valve CT calcium score thresholds for severe aortic stenosis have been proposed, validated,^11^^,^^12^ and recommended in recent international guidelines for the assessment of aortic stenosis severity when echocardiographic assessments are discordant.^13^ Mitral valve annular calcification is also a frequent finding on CCTA and may be associated with mitral valve dysfunction.^14^^,^^15^ Previous studies have suggested that patients with aortic stenosis and mitral valve calcification have an increased frequency of cardiovascular mortality beyond that related to the valve, including an increased risk of myocardial infarction (MI) and stroke, although the mechanisms underlying these associations remain unclear.^7^^,^^16^^,^^17^

In this study, we identified the prevalence and severity of incidental aortic and mitral valve calcification in patients undergoing CT as part of the Scottish COmputed Tomography of the HEART (SCOT-HEART) trial, and assessed their association with cardiovascular risk factors, coronary artery disease, and cardiovascular outcomes.

## Methods

### Study design

In the SCOT-HEART multicentre randomized controlled trial, 4146 patients with suspected angina due to coronary artery disease were randomized to undergo either standard care or standard care and CT.^18^The primary results of the SCOT-HEART trial have been published previously.^19–21^ Of the 1778 patients who subsequently underwent CT, 1769 images were available for analysis and included in this sub-study. Imaging included non-contrast CT for coronary artery calcium score and CCTA. The study was approved by the local ethics committee and participants provided written informed consent. The data underlying this article will be shared on reasonable request to the corresponding author.

### Demographic information and cardiovascular risk factors

Demographic information and information on cardiovascular risk factors were collected prospectively within the SCOT-HEART trial and obtained from the SCOT-HEART database.

### Computed tomography

CT imaging was performed using a 64 (Brilliance 64, Philips Medical Systems, Netherlands and Biograph mCT, Siemens, Germany) or 320 multidetector (Aquilion ONE, Toshiba Medical Systems, Japan) scanner. Non-contrast electrocardiogram-gated CT for coronary calcium scoring and contrast-enhanced CCTA were performed as described previously.^18^

### Assessment of valvular calcification

**Figure 1:**
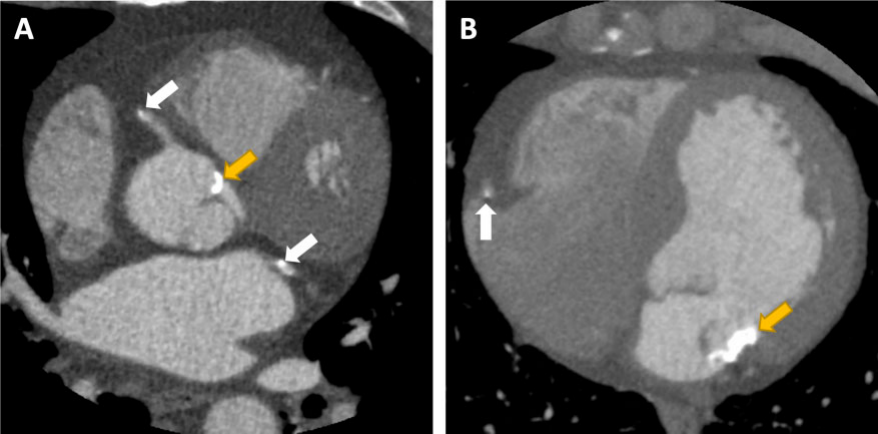
Calcification of the aortic (*A*) and mitral (*B*) annulus (yellow arrow in patients with co-existing coronary artery disease (white arrow).

All CCTA images were visually assessed for the presence of aortic or mitral valve/annulus calcification (*Figure 1*). Quantitative assessment of the aortic valve and mitral calcification was performed on non-contrast CT imaging using the Agatston scoring method as previously described.^12^^,^^15^^,^^22^Non-contrast images with 3-mm slices and 3-mm increment were assessed using semi-automatic software (VScore, Vital Images, USA). A threshold of 130 Hounsfield units was used for the identification of calcification. For cases where the location of the valve was uncertain on non-contrast images, contrast-enhanced CCTA was used to visually confirm the anatomy.

### Clinical outcomes

Outcome information was obtained from national electronic health linkage data provided by the electronic Data Research and Innovation Service (eDRIS) of the National Health Service (NHS) Scotland. Outcomes were confirmed by review of the patient health records where required. Surgical coding information was used to identify patients who underwent aortic or mitral valve surgery using the OPCS (Office of Population Censuses and Surveys) 4 Classification of Surgical Operations and Procedures.

### Statistical analysis

The primary composite endpoint of this sub-study was cardiovascular mortality, non-fatal MI, or non-fatal stroke. Quantitative data are presented as mean and standard deviation or, if the data were not normally distributed, median and interquartile intervals. Statistical significance was assessed using Pearson’s χ^2^ test, Fisher’s exact test, Students *t*-test, or Mann–Whitney *U* test, as appropriate. Correlations were assessed using Spearman’s rank order correlation (0–0.19 very weak, 0.2–0.39 weak, 0.40–0.59 moderate, 0.6–0.79 strong, and 0.8–1 very strong). Hazard ratios (HRs) and 95% confidence intervals (CIs) are presented. Ordinal regression analysis was performed to assess the effect of cardiovascular risk factors on aortic and mitral valve calcification. Multivariable analysis was performed including valvular calcification, coronary artery calcification, and the presence of obstructive coronary artery disease. Outcome data were analysed using Cox proportional hazards regression and presented graphically using cumulative incidence plots of the time to the first event. Statistical analysis was performed using R version 3.5.0 (R Foundation for Statistical Computing, Vienna, Austria). A statistically significant difference was defined as a two-sided *P*-value <0.05.

## Results

### Study population

Of the 4146 patients recruited into the study, 2073 were randomized to CT and 1769 CT scans were available and of suitable quality for analysis. Valvular calcification (*Table 1*) was identified in 273 (15%) patients, with aortic valve calcification occurring in 241 (14%) patients, mitral calcification in 64 (4%) patients, and combined aortic and mitral calcification in 32 (2%) patients. After 4.82 ± 1.13 years of follow-up, 53 patients had met the composite endpoint: cardiovascular mortality (*n* = 4), non-fatal MI (*n* = 38), or non-fatal stroke (*n* = 11).

**Table 1:** Characteristics of study participants

	All participants	Aortic valve calcification		Mitral valve calcification	
			*P*-value		*P*-value
N	1769	241 (14%)		64 (4%)	
Male	997 (56%)	178 (74%)	**<0.001**	41 (64%)	0.206
Age (years)	58 ± 9	64 ± 7	**<0.001**	65 ± 7	**<0.001**
Body mass index (kg/m^2^)	30 ± 6	30 ± 5	0.896	30 ± 5	0.386
Atrial fibrillation	34 (2%)	7 (3%)	0.213	2 (3%)	0.350
Smoking status					
Current smoker	330 (19%)	37 (15%)	**0.007**	5 (8%)	**0.007**
Ex-smoker	593 (34%)	102 (42%)		32 (50%)	
Non-smoker	845 (48%)	102 (42%)		27 (43%)	
Hypertension	608 (35%)	122 (51%)	**<0.001**	36 (57%)	**<0.001**
Diabetes Mellitus	196 (11%)	45 (19%)	**<0.001**	14 (22%)	**0.005**
Family history CHD	765 (45%)	91 (38%)	**0.048**	20 (32%)	0.053
Previous CHD	178 (10%)	39 (16%)	**<0.001**	11 (17%)	0.054
Previous PAD	31 (2%)	7 (3%)	0.178	1 (2%)	1
Previous CVD	79 (4%)	27 (11%)	**<0.001**	6 (10%)	0.059
Total cholesterol concentration (mg/dL)	192 ± 73	179 ± 74	**0.003**	182 ± 85	0.324
Anginal symptoms					
Typical angina	654 (37%)	124 (51%)	**<0.001**	33 (52%)	**0.046**
Atypical angina	432 (24%)	66 (27%)		11 (17%)	
Non-anginal	683 (39%)	51 (21%)		20 (31%)	
Risk score	17.9 ± 11.0	24.6 ± 11.1	**<0.001**	25.5 ± 11.5	**<0.001**

Data are presented as *N* (%), mean ± standard deviation, or median (interquartile range).

*P*-values represent comparison to total cohort.

CHD, coronary heart disease; CTCA, computed tomography coronary angiography; CVD, cerebrovascular disease; PAD, peripheral arterial disease. Bold values indicate statistical significance.

### Aortic valve calcification

**Figure 2:**
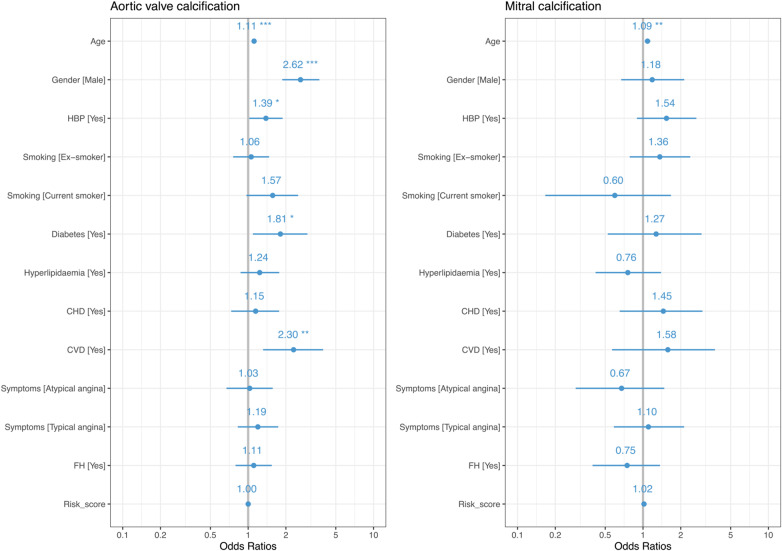
Multivariable analysis of cardiovascular risk factors and the presence of aortic valve or mitral calcification. Age (years); male gender compared to female gender; smoking status compared to non-smokers; total cholesterol concentration per unit increment; symptoms compared to those with non-anginal chest pain; Risk_score, ASSIGN cardiovascular risk score; AF, atrial fibrillation; BMI, body mass index; CHD, previous history of coronary heart disease; CVD, previous history of cerebrovascular disease; FH, family history of coronary heart disease; HBP, hypertension; PVD, previous history of peripheral vascular disease; *P < 0.05; **P < 0.01; ***P < 0.001.

Independent predictors of the presence of aortic valve calcification (*Figure 2*) were age [odds ratio (OR) 1.11; 95% CI 1.08–1.15; *P* < 0.001], male sex (OR 2.62; 95% CI 1.87–3.70; *P* < 0.001), hypertension (OR 1.39, 95% CI 1.02–1.88; *P* = 0.037), diabetes mellitus (OR 1.81; 95% CI 1.09–2.97; *P* = 0.020), and a prior history of cerebrovascular disease (OR 2.30; 95% CI 1.32–3.96; *P* = 0.003).

**Figure 3:**
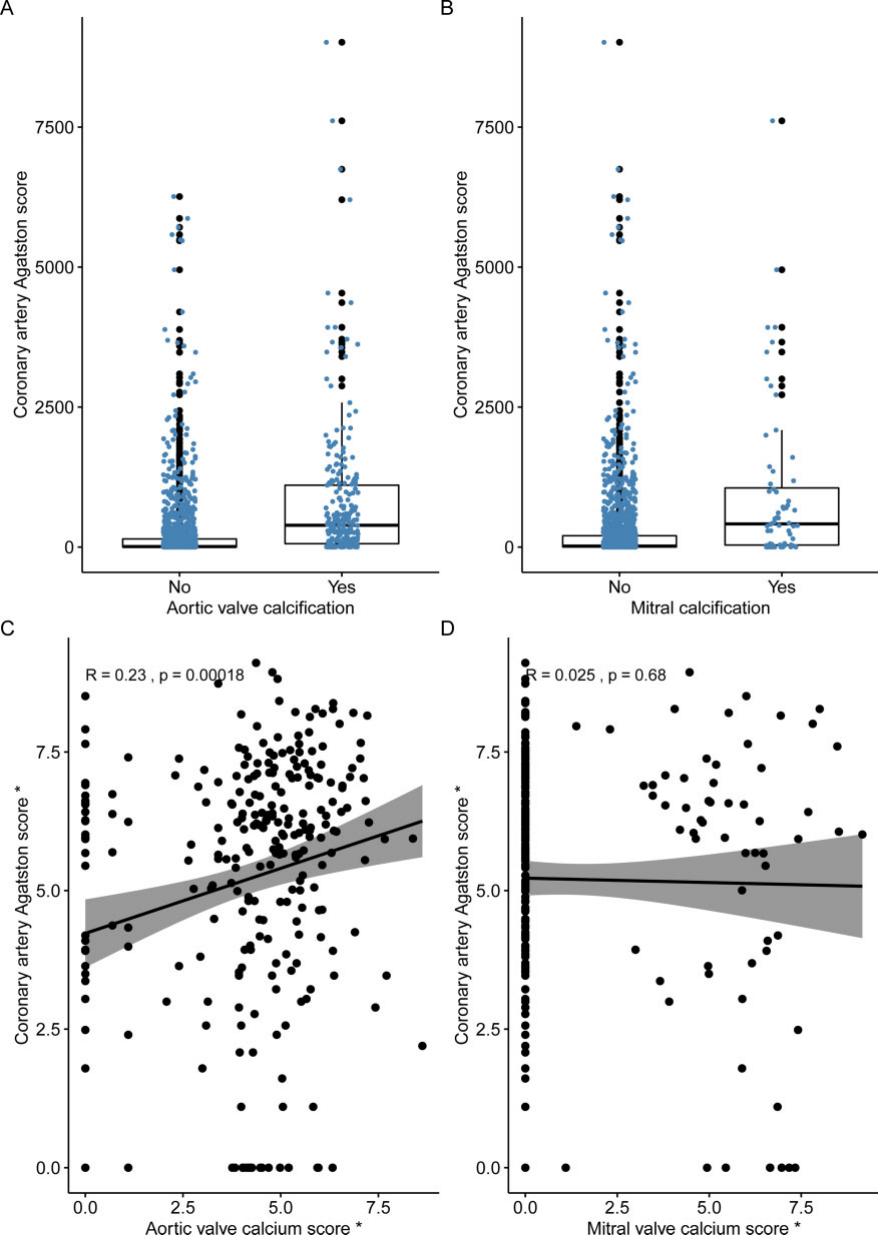
Coronary artery calcification was higher in patients with both (*A*) aortic valve calcification and (*B*) mitral valve calcification than patients without. Correlation between coronary artery Agatston calcium score and (*C*) aortic valve calcium score and (*D*) mitral valve calcium score. *log transformed.

Patients with aortic valve calcification had a higher coronary artery calcium score [390 (interquartile range, IQR 61–1105) Agatston units (AU)] compared to those without aortic valve calcification [8 (IQR 0–145) AU, *P* < 0.001, *Figure 3*]. Patients with aortic valve calcification were also more likely to have non-obstructive (41%) or obstructive (51%) coronary artery disease than patients without aortic valve calcification (*P* < 0.001). However, 9% (*n* = 21/241) of patients with aortic valve calcification had no coronary artery calcification, and 80% (*n* = 907/1127) of patients with coronary artery calcification had no aortic valve calcification. A correlation was observed between the aortic valve calcium score and coronary artery calcium score, but this was only weak (r = 0.23, *P* < 0.001, *Figure 3*).

**Figure 4:**
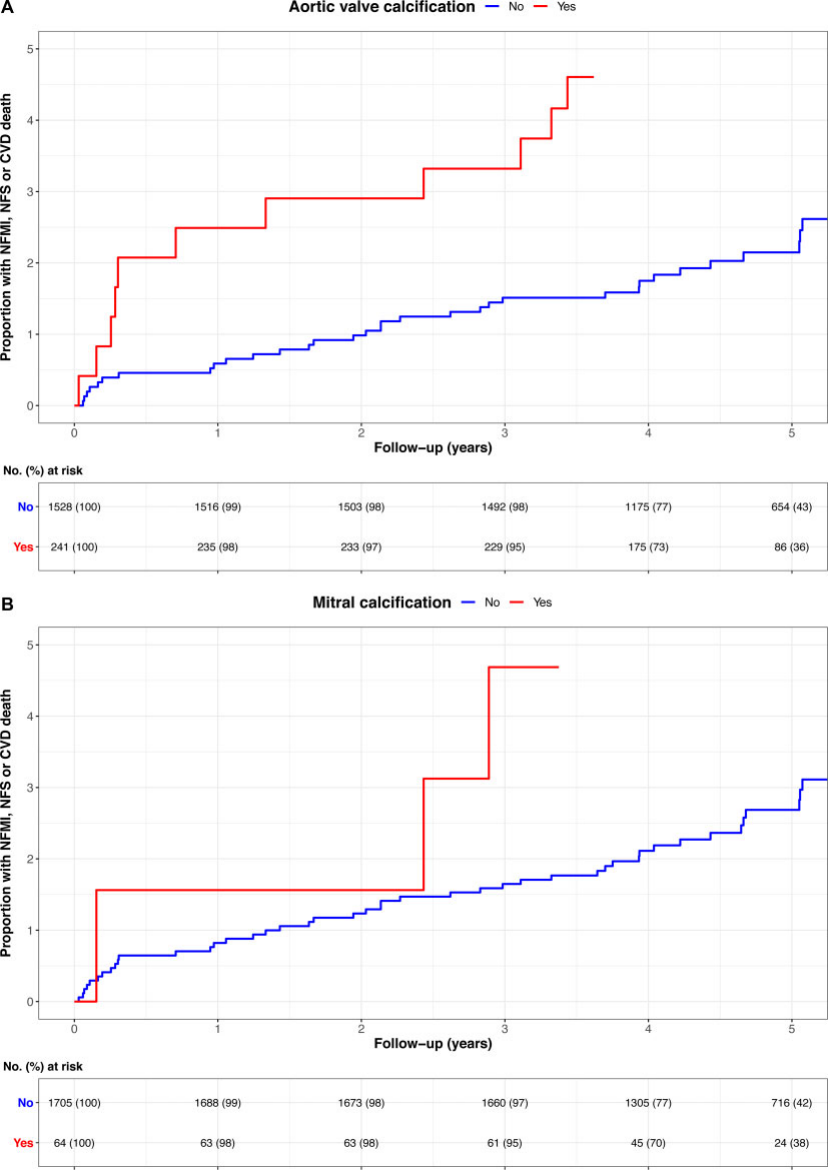
Cumulative incidence plot showing the association of the presence of (*A*) aortic valve calcification and (*B*) mitral calcification on the composite endpoint of cardiovascular death (CVD), non-fatal myocardial infarction (NFMI), or non-fatal stroke (NFS). The adverse outcomes associated with valve calcification were no longer evident once the burden of coronary atherosclerosis was considered in the multivariable model.

A higher proportion of patients with aortic valve calcification met the composite endpoint compared to patients without aortic valve calcification [6.6% (*n* = 16/241) vs. 2.4% (*n* = 37/1528), *P* = 0.002; HR 2.87; 95% CI 1.60–5.17; *P* < 0.001 (*Figure 4*)]. However, on multivariable analysis, only coronary artery calcium score was an independent predictor of the composite endpoint (*Table 2*).

**Table 2:** Univariable and multivariable analysis for aortic valve calcification and the composite endpoint of cardiovascular mortality, non-fatal myocardial infarction, or non-fatal stroke

	Univariable	Multivariable
Aortic valve calcification	2.87 (95% CI 1.60–5.17) *P* < 0.001	1.75 (95% CI 0.93–3.29) *P* = 0.081
Coronary artery calcification^a^	1.18 (95% CI 1.10–1.27) *P* < 0.001	1.12 (95% CI 1.02–1.22) *P* = 0.022
Obstructive coronary artery disease	2.94 (95% CI 1.72–5.04) *P* < 0.001	1.50 (95% CI 0.76–2.98) *P* = 0.241

CI, confidence interval.

a Per doubling.

### Mitral valve calcification

The only independent predictor of the presence of mitral valve calcification (*Figure 2*) was age (OR 1.09; 95% CI 1.03–1.15; *P* = 0.002). Patients with mitral calcification had a higher coronary artery calcium score [413 (IQR 36–1056) AU] compared to those without mitral calcification [18 (IQR 0–202) AU, *P* < 0.001, *Figure 4*]. Patients with mitral calcification were also more likely to have both non-obstructive (45%) and obstructive (44%) coronary artery disease compared to patients without (*P* < 0.001). However, 14% (*n* = 9/64) of patients with mitral calcification had no coronary artery calcification, and 95% (*n* = 1072/1127) of patients with coronary artery calcification had no mitral calcification. There was no correlation between mitral valve calcium score and coronary artery calcium score (*r* = 0.03, *P* = 0.68, *Figure 4*).

Patients with mitral valve calcification were more likely to meet the composite endpoint compared to patients without mitral valve calcification [9.4% (*n* = 6/64) vs. 2.8% (*n* = 47/1705), *P* = 0.01]: HR 3.50; 95% CI 1.47–8.07; *P* = 0.004 (*Figure 4*). However, on multivariable analysis only coronary artery calcium score was an independent predictor of the composite endpoint (*Table 3*).

**Table 3:** Univariable and multivariable analysis for mitral valve calcification and the composite endpoint of cardiovascular mortality, non-fatal myocardial infarction, or non-fatal stroke

	Univariable	Multivariable
Mitral valve calcification	3.45 (95% CI 1.50–8.07) *P* = 0.004	2.21 (95% CI 0.92–5.28) *P* = 0.075
Coronary artery calcification^a^	1.18 (95% CI 1.10–1.27) *P* < 0.001	1.13 (95% CI 1.03–1.24) *P* = 0.012
Obstructive coronary artery disease	2.94 (95% CI 1.72–5.04) *P* < 0.001	1.54 (95% CI 0.78–3.06) *P* = 0.214

CI, confidence interval.

a Per doubling.

## Discussion

In this large multicentre randomized controlled trial of patients undergoing CT for stable chest pain, we have shown that valvular calcification is a common incidental finding, occurring in one in six patients. Aortic and mitral valve calcifications are associated with traditional cardiovascular risk factors and underlying coronary artery disease. Whilst patients with aortic or mitral calcification were three times more likely to suffer an adverse cardiovascular event, this effect was not independent of the burden of coronary artery disease. This indicates that the adverse prognosis associated with valve calcification in these patients is mediated by its association with coronary artery disease, rather than representing a direct causal effect.

In our study, valvular calcification was associated with a three-fold increased risk of cardiovascular death, MI, or stroke, but this was not independent of coronary artery calcium score, a marker of atherosclerotic plaque burden. Similar results were reported in a study of lung cancer screening patients, in whom aortic valve calcification did not provide incremental prognostic information over coronary calcification.^23^ This is in contrast to previous studies which reported an association between aortic stenosis or mitral valve calcification and adverse cardiovascular outcomes. In the Cardiovascular Health Study (CHS) study of patients over 65 years old, the presence of aortic stenosis on echocardiography was associated with a 50% increase in the risk of cardiovascular death and MI.^24^ In the Multi-Ethnic Study of Atherosclerosis (MESA) primary prevention study, the presence of aortic valve calcification was associated with a 72% increased risk of MI and 50% increase in the risk of cardiovascular events.^25^ However, similar to our study, this effect was no longer statistically significant after adjustment for cardiovascular risk factors, inflammatory biomarkers and coronary artery calcium score.^25^ We therefore provide further evidence to support the theory that cardiovascular events in patients with valvular calcification are driven by the association with coronary artery disease, rather than the presence of valvular heart disease itself.

In this study, we have confirmed the close association between valve calcification and both cardiovascular risk factors and coronary atherosclerosis. Similar to previous studies,^15^^,^^26–28^ we found that age, male sex, hypertension, diabetes, and prior history of cerebrovascular disease were independent predictors of aortic valve calcification, whilst age was the only independent predictors of mitral calcification. This suggests that there are overlapping but distinct mechanisms underlying these pathologies.

The vast majority of patients with aortic and mitral valve calcification had evidence of co-existent coronary calcification on CT calcium scoring, reflecting the pathological overlap between atherosclerosis and the early stages of both aortic and mitral valve calcification, as well as shared risk factors for these conditions.^25^^,^^29–31^ However, the correlation between coronary artery calcification and aortic valve calcium scores was poor, and there was no correlation with mitral calcium scores. Although most patients with valvular heart disease had obstructive or non-obstructive coronary artery disease, few patients with coronary artery disease had valvular heart disease. Thus, the presence of valvular heart disease could be a trigger to assess the coronary arteries, but the presence of coronary artery disease is not an indication for valvular assessment with echocardiography.

Our study has several limitations. This includes the small number of clinical events and that patients were managed according to routine clinical practice rather than having management prescribed on the basis of the imaging findings. The prevalence of valvular calcification and cardiovascular risk factors varies depending on the cohort studied. The age range of the SCOT-HEART population meant that mild calcification was the most common pathology and that the 5-year duration of follow-up might not have been sufficient for this early stage of disease to result in clinical events. In addition, the SCOT-HEART participants were recruited from cardiology outpatient clinics with suspected angina due to coronary artery disease. Although two-thirds of the patients were ultimately diagnosed as not having angina due to coronary artery disease, these results may not be representative of asymptomatic patients or the general population. Finally, we do not have data on the progression of valve calcification in this cohort which in other studies appears unrelated to cardiovascular risk factors but is most closely associated with the severity of baseline valve disease and calcification.

## Conclusion

Valvular calcification is a frequent incidental finding on CCTA and is associated with cardiovascular risk factors and the presence of coronary artery disease. Whilst patients with aortic or mitral valve calcification demonstrate a three-fold increased risk of future adverse cardiovascular events, this appears to be mediated by co-existent coronary artery disease.

## Supplementary data

Supplementary data are available at *European Heart Journal - Cardiovascular Imaging* online.

## Funding

This trial was funded by the Chief Scientist Office of the Scottish Government Health and Social Care Directorates (CZH/4/588), with supplementary awards from Edinburgh and Lothian’s Health Foundation Trust and the Heart Diseases Research Fund. D.E.N. (CH/09/002, RG/16/10/32375, RE/18/10/33842) and M.C.W. (FS/11/014) are supported by the British Heart Foundation. M.C.W. is supported by the Chief Scientist Office of the Scottish Government Health (PCL/17/04). D.E.N. is the recipient of a Wellcome Trust Senior Investigator Award (WT103782AIA). P.D.A. is supported by a Heart Foundation of New Zealand Senior Fellowship (1844). A.J.M. is supported by a British Heart Foundation Accelerator Award Clinical Lectureship (AA/18/3/34220). E.v.B. is supported by the Scottish Imaging Network: A Platform of Scientific Excellence (SINAPSE). M.R.D. is supported by the British Heart Foundation (FS/14/78/31020) and the Sir Jules Thorn Biomedical Research Award 2015 (15/JTA). The Royal Bank of Scotland supported the provision of 320-multidetector CT for NHS Lothian and the University of Edinburgh. The Edinburgh Imaging facility QMRI (Edinburgh) is supported by the National Health Service Research Scotland (NRS) through National Health Service Lothian Health Board. The Clinical Research Facility Glasgow and Clinical Research Facility Tayside are supported by National Health Service Research Scotland (NRS).

**Conflict of interest:** Outside the submitted work, Professor Van Beek reports research support from Siemens and personal fees or other financial activity from QCTIS Ltd, Mentholatum, InHealth, and Aidence. All other authors have declared no conflict of interest.

## Supplementary Material

jeaa263_Supplementary_Data
