# Supplementary material for: Prevalence and clinical implications of valvular calcification on coronary computed tomography angiography
Source: Eur Heart J Cardiovasc Imaging. 2020 Dec 11;22(3):262–70. doi: 10.1093/ehjci/jeaa263 (PMC7899264; doi:10.1093/ehjci/jeaa263)
Supplement: jeaa263_Supplementary_Data [file jeaa263_supplementary_data.docx]

**Supplementary material**

*Supplementary Table i: Univariable analysis of risk factors for aortic and mitral calcification*

|  | | | Aortic valve calcification | | Mitral valve calcification | |
| --- | --- | --- | --- | --- | --- | --- |
|  |  |  | Odds ratio  (95% CI) | p | Odds ratio  (95% CI) | p |
| Age | | | **1.01 (1.01, 1.01)** | **<0.001** | **1.00 (1.00, 1.00)** | **<0.001** |
| Gender (Male) | | | **1.10 (1.07, 1.14)** | **<0.001** | 1.01 (1.00, 1.03) | 0.206 |
| BMI | | | 1.00 (1.00, 1.00) | 0.901 | 1.00 (1.00, 1.00) | 0.430 |
| Smoking | | Ex-smoker | **1.05 (1.02, 1.09)** | **0.005** | **1.02 (1.00, 1.04)** | **0.028** |
|  |  | Current smoker | 0.99 (0.95, 1.36) | 0.700 | 0.98 (0.96, 1.01) | 0.166 |
| Diabetes mellitus | | | **1.11 (1.06, 1.17)** | **<0.001** | **1.04 (1.01, 1.07)** | **0.005** |
| Hyperlipidaemia | | | **1.08 (1.04, 1.12)** | **<0.001** | 1.01 (0.99, 1.03) | 0.297 |
| Previous CHD | | | **1.10 (1.04, 1.16)** | **<0.001** | **1.03 (1.00, 1.06)** | **0.054** |
| Previous CVD | | | **1.24 (1.15, 1.34)** | **<0.001** | **1.04 (1.00, 1.09)** | **0.049** |
| Previous PVD | | | 1.10 (0.97, 1.24) | 0.139 | 1.00 (0.93, 1.06) | 0.916 |
| Symptoms | Atypical angina | | 1.02 (0.98, 1.06) | 0.307 | 1.00 (0.97, 1.02) | 0.739 |
|  | Typical angina | | **1.10 (1.06, 1.14)** | **<0.001** | 1.02 (1.00, 1.04) | 0.038 |
| Family history of CHD | | | **0.97 (0.94, 1.00)** | **0.049** | 0.98 (0.97, 1.00) | 0.053 |
| Atrial fibrillation | | | 1.07 (0.95, 1.21) | 0.232 | 1.02 (0.96, 1.09) | 0.476 |
| Cardiovascular risk score | | | **1.01 (1.01, 1.01)** | **<0.001** | **1.00 (1.00, 1.00)** | **<0.001** |

*Supplementary Table ii: Prevalence and severity of coronary artery calcification and coronary artery disease on CCTA in patients with aortic valve calcification and mitral valve calcification.*

|  | | **All participants** | **Aortic valve calcification** | | **Mitral valve calcification** | |
| --- | --- | --- | --- | --- | --- | --- |
|  |  |  |  | p value |  | p value |
| N | | 1769 | 241 (14%) | - | 64 (4%) | **-** |
| Coronary artery calcium score (Agatston units) | | 21 [0, 230] | 388 [61, 1091] | **<0.001** | 413 [36, 1056] | **<0.001** |
| CCTA | Normal | 646 (37%) | 18 (7%) | **<0.001** | 7 (11%) | **<0.001** |
|  | Non-obstructive | 671 (38%) | 100 (41%) |  | 29 (45%) |  |
|  | Obstructive | 452 (26%) | 123 (51%) |  | 28 (44%) |  |
